# A Review of Laser Ultrasonic Lamb Wave Damage Detection Methods for Thin-Walled Structures

**DOI:** 10.3390/s23063183

**Published:** 2023-03-16

**Authors:** Shanpu Zheng, Ying Luo, Chenguang Xu, Guidong Xu

**Affiliations:** 1 Faculty of Civil Engineering and Mechanics, Jiangsu University, Zhenjiang 212013, China; 2 School of Physics and Electronic Engineering, Jiangsu University, Zhenjiang 212013, China

**Keywords:** thin-walled structures, nondestructive testing, laser ultrasonic detection, ultrasonic lamb wave, damage imaging algorithm

## Abstract

Thin-walled structures, like aircraft skins and ship shells, are often several meters in size but only a few millimeters thick. By utilizing the laser ultrasonic Lamb wave detection method (LU-LDM), signals can be detected over long distances without physical contact. Additionally, this technology offers excellent flexibility in designing the measurement point distribution. The characteristics of LU-LDM are first analyzed in this review, specifically in terms of laser ultrasound and hardware configuration. Next, the methods are categorized based on three criteria: the quantity of collected wavefield data, the spectral domain, and the distribution of measurement points. The advantages and disadvantages of multiple methods are compared, and the suitable conditions for each method are summarized. Thirdly, we summarize four combined methods that balance detection efficiency and accuracy. Finally, several future development trends are suggested, and the current gaps and shortcomings in LU-LDM are highlighted. This review builds a comprehensive framework for LU-LDM for the first time, which is expected to serve as a technical reference for applying this technology in large, thin-walled structures.

## 1. Introduction

Metal/composite thin-walled structures are widely used as an essential load-bearing structure in large equipment, such as aircraft skins [1,2], ship shells [3], and energy storage tanks [4,5]. Large thin-walled structures are typically several meters in size but only a few millimeters thick [6,7]. During service, these structures are subjected to complex and alternating loads for extended periods, or to sudden impacts. Structures that exceed their design life are also susceptible to performance degradation. The above factors may lead to imperceptible damage, such as delamination and debonding in composites [8], or corrosion and impact in metals [9]. Therefore, the development of a robust NDT method is imperative to detect damage during the manufacturing stage, as well as to perform routine maintenance during the operational stage [10].

When the ultrasonic wave length is approximately the same dimension as the geometric size of the structure, the ultrasonic guided wave is formed by the superposition of interference and dispersion after the wave is reflected repeatedly in the structure [11]. For free boundary plates, with zero stress on the upper and lower surfaces, the guided waves propagating in them are called ultrasonic Lamb waves. According to the different vibration modes, ultrasonic Lamb waves are divided into symmetric and anti-symmetric modes. Thin-walled structures are typical plates/shells in which ultrasonic Lamb waves can propagate over long distances with low energy attenuation. Structural anomalies change a structure’s local/global properties, and ultrasonic Lamb wave detection aims to capture the dynamic response of the structure (e.g., scattered waves, vibration mode) and then interpret the physical properties [12]. Laser ultrasonics is a non-contact sensing technique. When a high-energy pulsed laser is incident at a point on the structure surface, then the laser is absorbed to a certain depth [13,14]. The release of heat energy causes local expansion, resulting in local stress and strain as the source of waves, so that waves propagate within and on the surface. Finally, the wavefield is collected and used as the basis for analyzing structural anomalies [15]. The generation mechanism of laser ultrasound is either thermoelastic (nondestructive) or thermal etching (destructive), and this paper focuses on the former [16].

The strengths of LU-LDM lie in its ability to perform online inspection, as demonstrated by the following aspects. Firstly, the technology facilitates long-distance operation, positioning detection systems from tens of centimeters to several meters away from the structure [17]. This means structures with higher positions and larger areas, such as aircraft wings, can be inspected. Secondly, the technique allows non-contact operation, which avoids many inconveniences of the contact/embedded sensor technique [18]; for example, couplant contamination, welding line, calibration and maintenance. Thirdly, with the assistance of the galvanometric laser mirror scanner (robot2) or multi-degree-of-freedom manipulator for laser deflection, as well as the development of machine vision technology and upper computer software, the distribution of laser measurement points in space has high designability [19,20,21]. Finally, the sensing technology, based on a laser vibrometer, has high accuracy and high scanning density, which can provide high-resolution and wavefields. In conclusion, LU-LDM satisfies the need for non-contact, long-distance, and on-site inspection of large thin-walled structures. Moreover, it offers high design flexibility and measurement accuracy.

In the last decade, contact-based sensing techniques for ultrasonic Lamb wave damage detection have been fully developed. However, LU-LDM differs from these techniques in several aspects, such as Lamb wave modes, bandwidth, and hardware equipment. A unique framework has been established for LU-LDM in this review, which combines the characteristics of laser ultrasound technology with ultrasonic Lamb wave detection technology. Extensive research showed that the review studies in the past 20 years have focused on contact ultrasonic Lamb wave damage detection methods [11,22,23] and the application of laser ultrasonic detection techniques [24,25,26,27]. However, to the best of our knowledge, no relevant review has comprehensively analyzed the application of LU-LDM in thin-walled structures. This review presents a comprehensive framework of LU-LDM based on research conducted over the past 20 years. The displacement amplitude, frequency, wave number, arrival time, and other characteristics of Lamb waves in laser ultrasonic testing can be used as feature parameters for evaluating the damage. Different parameters represent different physical meanings and are worthy of further research.

The rest of this paper is organized as follows. Section 2 summarizes five characteristics of the LU-LDM. Section 3 identifies the technical framework of the LU-LDM order of this review. Section 4 and Section 5 provide a summary of methods based on full wavefield data and a small amount of wavefield data. Section 6 lists four combination types to enhance the overall detection efficiency and precision. Section 7 outlines several future perspectives for the technology. In the last section, there is a summary.

## 2. Characteristics of LU-LDM

The excitation, propagation, and acquisition characteristics serve as the theoretical basis of LU-LDM. This section provides a summary of the characteristics of LU-LDM based on these aspects.

(1) Broadband characteristics

**Figure 1:**
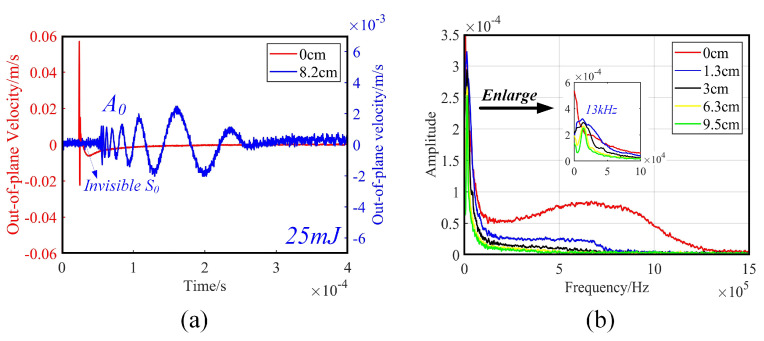
Time domain (*t*-domain) and frequency domain (*f*-domain) characteristics of laser ultrasonic Lamb wave. (**a**) Response signals at 0 and 8.2 cm from the excitation source. (**b**) Frequency spectrum of the response signal at 0 to 9.5 cm from the excitation source.

Using a 1 mm aluminum plate as an example, the laser ultrasonic detection system was used to excite ultrasonic Lamb waves. The wavefield signals of uniformly distributed and discretized points at different distances from the excitation point were collected to form a dense line scan of the wave field. This section analyzes the characteristics of laser ultrasonic Lamb waves in the *t*-domain and *f*-domain. As shown in Figure 1a, the red waveform collected at the center of the excitation source exhibited a unipolar Gaussian pulse with uneven frequency distribution in the range of 0–1 MHz. As the propagation distance increased, the Lamb wave evolved into an oscillatory signal with a gradually diminishing frequency. The frequency components near the excitation source were broad, the high frequency decayed rapidly, and the frequency spectrum gradually concentrated below 100 kHz at about 10 cm away from the excitation source, as shown in Figure 1b.

The bandwidth of laser ultrasound can be altered depending on the time and space. A cylindrical planoconvex lens was applied to transform the laser spot into a line source, which alters the directivity of the wavefield [28,29]. We utilized acoustic lenses, slit masks and an optical interferometer to excite narrow-band ultrasonic Lamb waves, and used the wavelength matching method to adjust the array element spacing to achieve frequency selection [30,31].

(2) Low-order anti-symmetric mode

**Figure 2:**
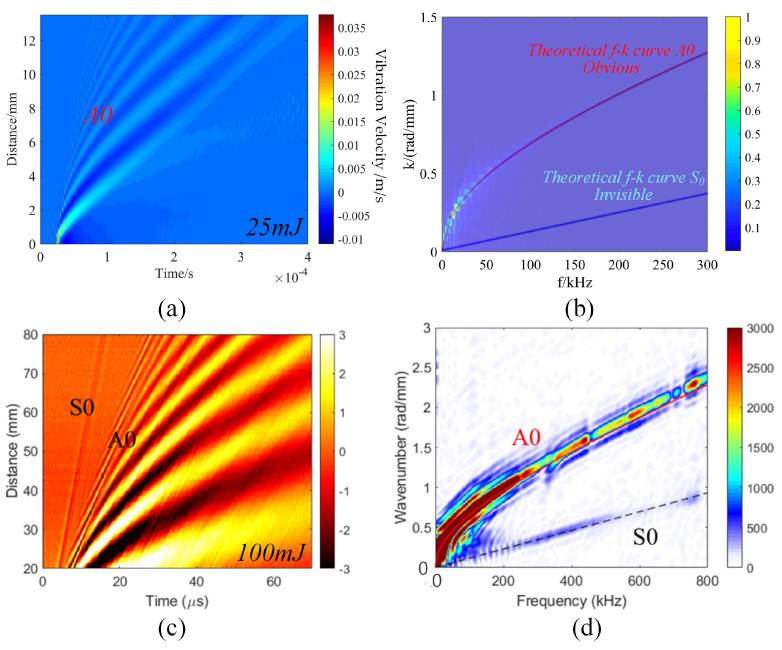
(**a**) Time-space (*t*-*s*) wavefield and (**b**) frequency-wavenumber domain (*f*-*k* domain) maps of ultrasonic Lamb waves excited by a laser at a single pulse energy of 25 mJ. (**c**) *t*-*s* wavefield and (**d**) *f*-*k* domain maps of ultrasonic Lamb waves excited by laser at a single pulse energy of 100 mJ [32].

As shown in Figure 2a,b, when the single pulse energy of the excitation laser was 25 mJ, in the *t*-*s* wavefield and *f*-*k* domain, the A${}_{0}$ mode was prominent, but the S${}_{0}$ mode was barely visible. In contrast, Figure 2c,d shows that when the energy was 100 mJ, a distinct A${}_{0}$ mode and a faint S${}_{0}$ mode could be observed, indicating that out-of-plane displacement could be enhanced by increasing pulse energy [32]. However, surface ablation occurs at this time, and it is necessary to ensure that the material surface is not ablated. Therefore, the laser power density cannot be higher than the ablation threshold, which is about 10${}^{7}$ W/cm${}^{2}$. From the analysis of the frequency components in Figure 1b, it is clear that the main energy was concentrated below 100 kHz. The A${}_{0}$ of the ultrasonic Lamb wave was dominated by out-of-plane displacement. The laser interferometry principle determines that it is more sensitive to out-of-plane displacements [33]. Therefore, the low-order anti-symmetric mode A${}_{0}$ was mainly analyzed in LU-LDM of a 1 mm aluminum plate.

(3) Limitations: low signal-to-noise ratio

The measurements of laser ultrasonic Lamb waves are susceptible to interference from external environmental factors, such as vibration and temperature [34]. The surface state of the structure also has a significant impact on the detection result. The above factors contribute to a low SNR in the laser ultrasound signal. Consequently, it is more challenging to extract valid scattering information from the signal. The most straightforward method for noise reduction is to place reflective tape in the area surrounding the measuring point. Metal structures are typically coated and polished, making their surfaces smooth and requiring high precision in the perpendicularity of the incident sensing laser. Minor angular deviations can impede the reflection of the laser back to the vibrometer for interference with the reference laser, leading to a low SNR. Thus, pasting silver-plated reflective tape is often necessary to create diffuse conditions in the detection region, effectively enhancing the SNR. It is worth noting that, for the plate with a thickness of less than 1 mm, the behavior of pasting reflective tape impacts the wavefield measurement, and the original surface should be used as far as possible. Enhancing the pulse energy of the excitation laser is also the most direct method of noise reduction, but the ablation threshold limits it. Therefore, aluminum foil can be pasted on the excitation point to protect the structure’s surface [35].

**Figure 3:**
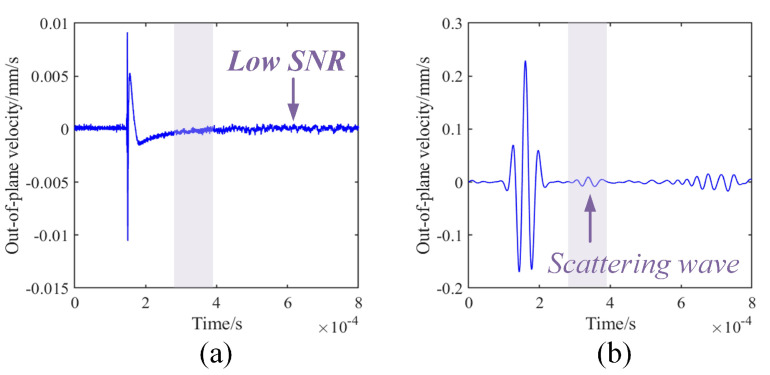
Limitation of low signal-to-noise ratio (SNR) of laser ultrasonic Lamb wave. (**a**) Scattered waves are submerged. (**b**) Scattering waves are highlighted after the continuous wavelet transform (CWT).

Since the amplitude level of the scattered wave is typically smaller than that of the direct wave, identifying and extracting parameters can be challenging. Noise reduction can be achieved through methods such as multiple averaging measurements or utilizing filtering algorithms. Usually, continuous wavelet transform (CWT) is used to extract the components at a specific frequency, which has the most significant correlation with the intensity of the scattering wave [36,37]. As shown in Figure 3, through-hole damage, with a 0.8 cm diameter, located 10 cm away from the excitation point was created, and the damage scattering wave was collected 2 cm to the right of the excitation point. The narrowband component was extracted using a wavelet packet with a center frequency of 120 kHz, and the scattering wave was significantly enhanced. In addition, the latest technology has developed a transducer with high photoacoustic conversion efficiency, which can be attached to the structure’s surface. The laser incident on the transducer can stimulate strong ultrasonic amplitude while protecting the surface [38].

(4) Hardware configuration with flexibility

Laser ultrasound is usually generated by solid lasers (such as Nd: YAG) and gas lasers (such as CO${}_{2}$). Q-switched/mode-locking techniques can obtain ultrashort laser pulses with narrow pulse widths and high peak power. The pulse duration for laser ultrasound detection is usually chosen to be 5–10 ns. The body wave excited by the pulse laser has a broadband characteristic with a non-uniform frequency distribution from zero to several MHz. By theoretical calculations [13], in the 1 mm aluminum plate, the maximum body wave frequency that can be excited is 19 to 37 MHz. The main frequency of an ultrasonic Lamb wave used for detection is generally not more than 1MHz. Therefore, pulse duration between 5–10 ns is suitable to LU-LDM in thin-walled structures.

As for wavefield sensing, the most widely used technique is the double-beam heterodyne interferometer, based on the Doppler effect, commonly referred to as the laser Doppler vibrometer (LDV) [26]. Compared with other intensity modulation interferometers, it has higher sensitivity and stability. The Confocal Fabry–Perot Interferometer has a poor response at low frequencies. The surrounding environmental vibration does not affect the device. Therefore, it is more suitable for application in the complex environments of the industrial field. Nevertheless, the main drawback is that it is not highly sensitive to vibrations [39]. The Photorefractive Two-Wave Mixing Interferometer has better sensitivity at low frequencies and can, therefore, more easily detect materials with strong attenuation. This setup is well-suited for applications in multiplexing schemes, where a surface is projected with an array simultaneously, resulting in substantial increases in detection speed [40].

According to the acoustic reciprocity theorem [41], Laser ultrasonic detection can be conducted through either fixed-point excitation-scanning measurement or scanning excitation-fixed-point measurement. The latter only needs to collect at a fixed point and paste local reflective tape. Therefore, the latter has relaxed the requirements for the incidence angle. The incidence angle of the excitation laser can reach $70^{\circ }$, which applies to the detection of curved structures [42]. Given the progressiveness of the scanning laser doppler vibrometer (SLDV), most studies still use the former detection mode.

**Figure 4:**
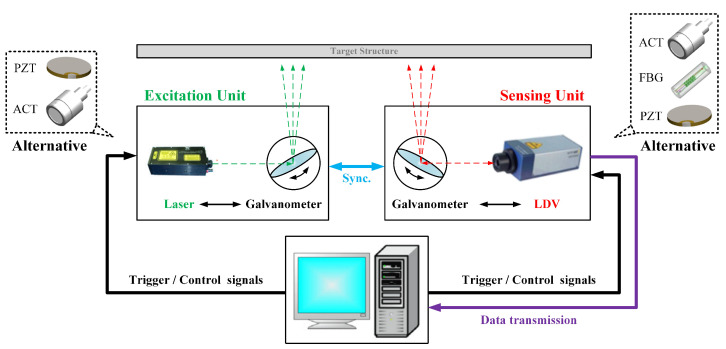
Setup of the laser ultrasonic Lamb wave detection system.

The full non-contact laser ultrasonic detection system mainly comprises two sets of equipment: the pulse laser and the laser vibrometer. The laser vibrometer is triggered externally by the pulsed laser, synchronously. The wavefield scanning speed depends on the repetition rate of the laser, which is limited by the residual guided wave excited last time. Due to the different attenuation characteristics, the repetition frequency of laser excitation used for metal and composite materials is 100 Hz and 1.5 kHz [43]. In laser ultrasonic detection systems, the transmitting and receiving devices are not integrated, and the instrument configuration is flexible. The laser vibrometer can cooperate with an air-coupled transducer (ACT) and a lead zirconate titanate transducer (PZT), and the laser can cooperate with PZT, ACT and fiber Bragg grating (FBG) sensor [18,44,45,46]. In addition, a new air-coupled optical microphone is also used as a vibrometer to cooperate with the laser [47]. The specific implementation can be configured according to specific conditions, as shown in Figure 4.

(5) Exact laser vibration measurement

Thanks to the assistance of laser deflection devices, such as the LMS or multi-degree-of-freedom manipulator, LU-LDM can precisely control the angle and distance of the laser [21]. The exact wavefield acquisition is reflected in three aspects. For one thing, the spot diameter of the laser can reach several hundred microns, most of the moving steps are set to 1–2 mm, and a moving step of 50 μm can achieve realistic fatigue closure cracks in microstructures [48]. Secondly, the point source has a high spatial position precision, directly affecting the damage localization precision based on the time of flight (TOF) [49]. Third, the vibrometer has a micron-level resolution of out-of-plane velocity several meters away. The SLDV from Polytec (Germany), a resolution of sensing out-of-plane vibration velocity can reach 20 nm/s, far exceeding the velocity 500 μm/s of an ultrasonic Lamb wave.

In conclusion, the laser ultrasonic Lamb wave is a broadband signal. The tool for characterizing the damage is mainly the low-order anti-symmetric mode A${}_{0}$ with a wavelength of 10–20 mm, but the large wavelength limits the detection resolution. Dispersion and low SNR can impede the effective extraction of scattering information, necessitating algorithms or devices to enhance the scattering wave. In terms of hardware configuration, the laser and vibrometer can be flexibly combined with other sensors.

## 3. Classification Criteria and Overall Framework

**Figure 5:**
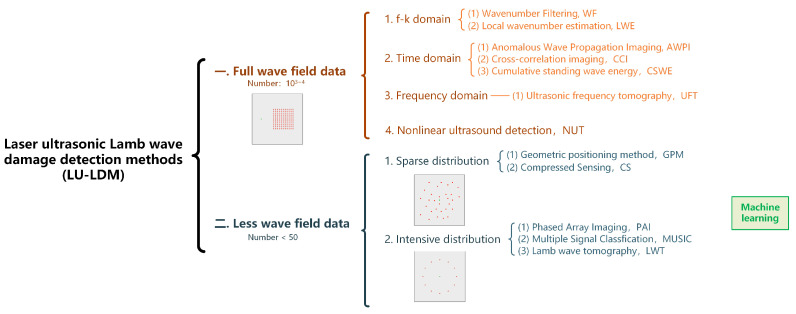
The overall framework of LU-LDM.

International institutions have recently conducted much LU-LDM research around thin-walled structures. According to the number of wavefield acquisitions, LU-LDM can be divided into two categories: methods based on full wavefield data and methods based on a small amount of wavefield data. These methods can be further classified based on the spectral domain type and the measurement points’ distribution density. Figure 5 shows the overall framework, which is also the outline of this overview.

Detection methods based on full wavefield data benefit from spatially dense scanning. They can characterize damage details, including depth and morphology, and have high imaging resolution. Scanning a specific area at intervals of a few millimeters can collect up to $10^{3∼4}$ data points. The collected three-dimensional matrix represents the *t*-*s* wavefield of the ultrasonic Lamb wave. Based on the spectral domain type of signal processing methods, several detection methods are classified into the *t*-domain, *f*-domain and *f*-*k* domain.

Based on the distribution density of the measurement points, the detection methods based on a small amount of wavefield data are divided into sparse distribution and dense distribution. Dense distribution requires that the spacing of measurement points is equivalent to the wavelength, and requires a particular topological array. Sparse distributions have large and irregular intervals between measurement points. A small amount of wavefield data (usually less than 50) is used to invert the damage location and size. Since the wavefield data from sparse distributions is incomplete, accurately locating damage can be challenging for most methods. As a result, the estimated location of the damage could be more precise.

## 4. Detection Method Based on Full Wavefield Data

### 4.1. Detection Methods in the *f*-*k* Domain

#### 4.1.1. Wavenumber Filtering

**Figure 6:**
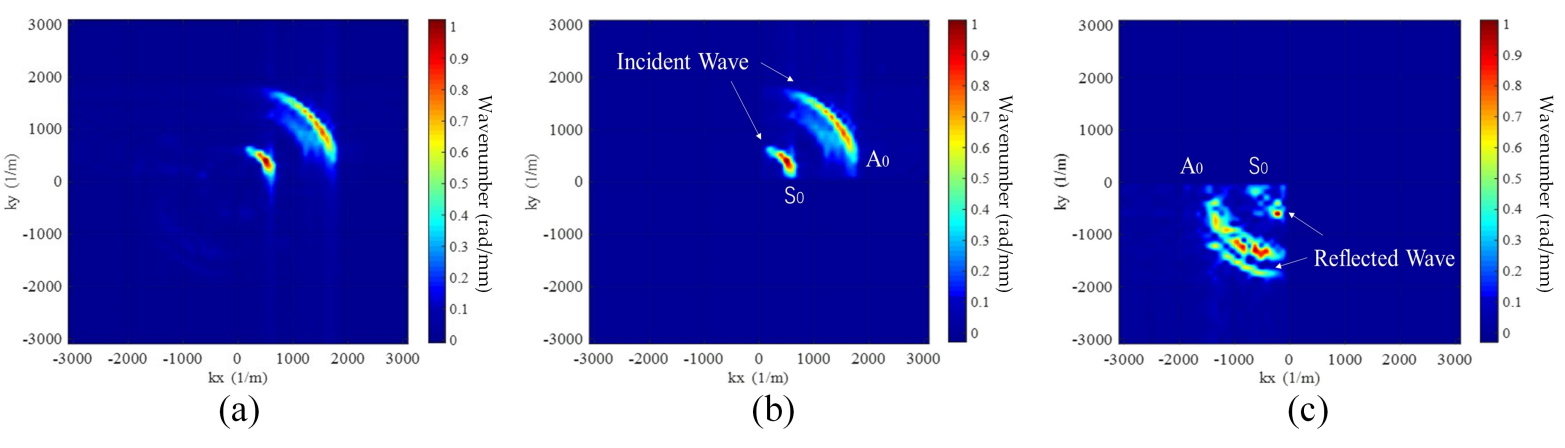
Wavenumber domain information and window function are used to separate incident and reflected waves. (**a**) Original wavenumber domain information. (**b**) The incident wavefield. (**c**) The reflected wavefield.

The complex propagation mechanism of ultrasonic waves and the multimodal characteristic of Lamb waves are the main factors hindering signal processing [50]. Wavefields that cannot be separated in the time domain can be separated in the *f*-*k* domain. The Wavenumber Filtering (WF) algorithm is used to convert the wavefield data at a specific time from the *t*-*s* domain to the *f*-*k* domain using the two-dimensional Fourier transform [51]. The wavefield in different directions/modes is separated using a window function, as shown in Figure 6, and then converted back to the *t*-*s* domain by the inverse Fourier transform. M. Ruzzene et al. [52] utilized WF to directly filter incident waves to highlight the scattering wavefield with damage. This approach detected narrow grooves in aluminum plates and debonding in tongue-and-groove joints.

Acquiring data from only a single incidence direction can provide only partial information on the damage boundary. Ma et al. [53] obtained more information by increasing the angle of incidence. Multiple image fusion methods were used to detect various types of damage, such as a triangular notch in an aluminum plate and the shape of letters produced by 3D printing technology. In addition, WF is also used in damage detection of the small amount of wavefield data. Jeon et al. [54] gathered wavefield data from a square ring array and highlighted the damaged scattered waves by WF. Many methods, described later, require pre-extraction of the wavefield in a specific direction/mode. Therefore, it is necessary to introduce the principle of WF in advance.

#### 4.1.2. Local Wavenumber Estimation

**Figure 7:**
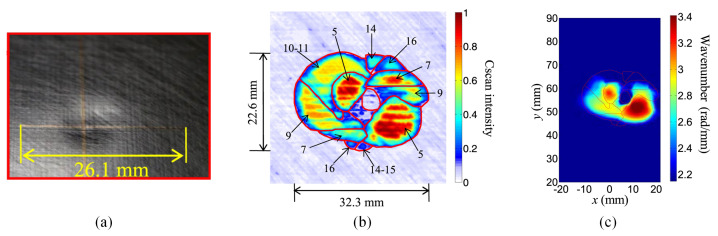
Comparison of composite delamination and imaging results [57]. (**a**) Shape of impact damage. (**b**) C-scan imaging (reference). (**c**) LWE imaging.

When an ultrasonic Lamb wave propagates at a specific frequency, its wavenumber is a definite function of material thickness, which allows the assessment of damage depth to be based on this function. The local wavenumber estimation (LWE) algorithm converts the three-dimensional *t*-*s* matrix of the full wavefield into the *f*-*k* domain through a three-dimensional Fourier transform [55]. The *f*-*k* domain contains spatial wavenumber information at different frequencies. This method averages the signal strengths within a specific frequency range. These wavenumbers are significantly higher at the thickness reduction than in the surrounding area, indicating the presence of damage [56]. Tian et al. [57] achieved the visualization of delamination, due to impact within the composite, through the LWE algorithm. The shape of the damage in the wavenumber distribution map was highly similar to the shape of the shallower parts in C-scan imaging, as shown in Figure 7. He also pointed out that the LWE algorithm is insensitive to deeper delamination in a previous report.

By adjusting the incident angle to excite a single-mode ultrasonic Lamb wave, the groove damage located at different depths of aluminum/CFRP plates was visualized, and the shape of the damage was identified [58]. Previous LWE algorithms only analyzed narrowband wavenumber information. In contrast, Gao et al. [59] took full advantage of the broadband characteristic of laser ultrasonic waves and used wavenumber information at multiple frequencies to optimize the damage details. High-accuracy imaging of circular delamination in aluminum plate bonded parts was achieved by data fusion.

The LWE is a high-precision imaging algorithm with the highest application rate in recent years. Its principle determines that it is more sensitive to damage with reduced thickness, such as composite delamination, aluminum alloy corrosion and notch.

### 4.2. Detection Methods in the *t*-Domain

#### 4.2.1. Anomalous Wave Propagation Imaging

As a laser has stable excitation, the generated ultrasonic field is also stable, so an abnormal wave field can be detected. These wavefields may consist of scattered and confined waves with standing wave characteristics [1]. In the scanning excitation–fixed-point measurement method, the waveforms of two adjacent points are very similar, and the wavefield at a damaged location is significantly different from that at an undamaged position [4]. During anomalous wave propagation imaging (AWPI), the first step is to match the arrival time and amplitude of adjacent signals. Then, the two are subtracted to suppress the incident wave and amplify the anomalous wave. Finally, a variable time window is proposed so that the damage imaging is closer to the actual damage. Lee et al. [1] utilized the AWPI method to successfully visualize impact damage in CFRP composite wings and to sequentially assess the length of cracks in the lining of aluminum alloy fuel tanks.

AWPI relies heavily on fast laser scanning and advanced computational capabilities. AWPI does not require complex theoretical knowledge or conversion between spectral domains. Instead, it relies on a relatively simple *t*-domain computation, making it well-suited for evaluating defects in complex structures. This approach holds great promise for achieving real-time defect detection in the field, with the added benefit of automated results processing.

#### 4.2.2. Cross-Correlation Imaging

The principle of 2D cross-correlation imaging (CCI) is that the incident and time-reversed scattered waves coincide at structural discontinuities [60]. The algorithm requires the incident wavefield $v_{incident}$(x,y,t) and the scattered wavefield $v_{scattered}$(x,y,t) to be extracted separately by WF. A two-dimensional correlation calculation is performed for the two wavefields, as shown in Equation (1) [61].

(1) $$\begin{array}{c} I\left(x,y\right)=\int_{0}^{T}v_{\mathrm{incident}\;}\left(x,y,t\right)v_{\mathrm{scattered}\;}\left(x,y,t\right)dt \end{array}$$

The intercorrelation values at the damage are much larger than those at the undamaged locations. Xiao et al. [61] accurately quantified cracks in aluminum specimens by the CCI method with an error of only 3.3%. The stainless steel specimens were monitored for crack extension under fatigue loading with quantification errors as low as 2% for 5 mm cracks. In a subsequent study [44], they constructed a fully non-contact detection system with ACT-SLDV to achieve high-precision imaging of notches in thin stainless steel plates with varying lengths, depths, and orientations. He et al. [62] proposed innovative imaging conditions which can compensate for the attenuation effect due to distance. Quantitative imaging of a composite impact was achieved, which provided enhanced imaging of multi-site damage.

CCI takes advantage of the time-reversal symmetry of the fluctuation equation, enabling the automatic compensation of dispersion effects. However, this method requires significant computational amount.

#### 4.2.3. Cumulative Standing Wave Energy

Reflected waves are generated near the damage, and the incident and reflected waves are superimposed on each other to form interference waves. Since the excitation signal is transient, the interference is also not stable. The instantaneous power can characterize the energy of the signal. The instantaneous power of the wavefield not only contains the incident and reflected waves but also superimposes the interfering waves [63]. Using WF to extract the scattered and incident waves, the integral of the difference between the measured wavefield energy minus the incident and reflected wavefield energies is the cumulative standing wave energy (CSWE), and the imaging principle is given in Equations (2) and (3) [64].

(2) $$\mathrm{SWE}\left(x,t\right)=W_{T}^{2}\left(x,t\right)-W_{F}^{2}\left(x,t\right)-W_{B}^{2}\left(x,t\right)$$

(3) $$\mathrm{CSWE}=\int_{0}^{t}\mathrm{SWE}\left(x,t\right)dt$$

The damage size can be analyzed through standing wave energy distribution maps. Zhang et al. [65] achieved the length visualization of slender notches in aluminum plates. H. Sohn et al. [64] achieved the visualization of standing wave energy induced by cracks, and the crack length was effectively evaluated by standing wave energy. The CSWE imaging principle is relatively straightforward, and the computational requirements are not excessive, enabling the assessment of damage sizes at the centimeter-level.

### 4.3. Detection Methods in the *f*-Domain

**Figure 8:**
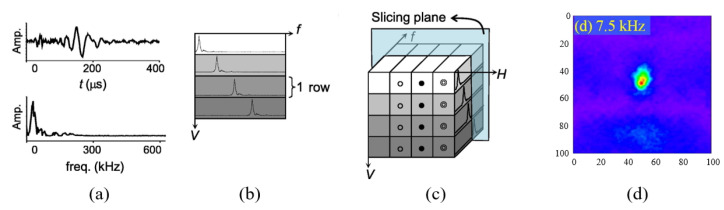
Principle and results of UFT imaging of impact damage in radome. (**a**) Characteristics of Lamb waves in *t*-domain and *f*-domain [66]. (**b**) Signals in the *t*-domain of vertical scan. (**c**) Selected tomogram along the frequency axis. (**d**) Tomogram at a frequency of 7.5 kHz.

As the incident wave propagates to the damage, it produces a new frequency component or offset. Ultrasonic frequency tomography (UFT) is based on the principle that a Fourier transform of the *t*-domain signal at each scan point constitutes a new three-dimensional matrix. At each frequency, the tomogram displays the distribution of that frequency at various locations, as shown in Figure 8. In order to pinpoint the location of damage, it is necessary to determine the frequency range associated with the damage in advance. The tomogram at that specific frequency can then be selected as the damage assessment map. Lee et al. [66] detected circular impact damage in a glass fiber composite plate of a honeycomb sandwich radome, and its location and size matched well with the actual impact damage. In a further study, they [48] built the ACT-SLDV non-contact nondestructive detection system, by setting the laser scan interval to 50 μm. The quantitative evaluation of real fatigue microcracks was achieved with a size detection accuracy of 96%.

UFT does not require reference signals and can be used as a rapid imaging algorithm after a laser ultrasonic Lamb wave C-scan, which makes it ideal for applications that require real-time imaging capabilities.

### 4.4. Nonlinear Ultrasound Detection Method

Linear ultrasound detection methods, based on reflection, diffraction, attenuation, or mode transformation after the interaction between Lamb wave and damage, are typically not sensitive to microdamage such as microcracks and pores. Nonlinear ultrasound detection (NUT) methods rely on the harmonic, subharmonic, or modulated waves generated by the interaction between ultrasonic waves and micro damage [67]. In summary, nonlinear ultrasound is a powerful tool for the detection of early damage, and when it is combined with LU-LDM, it has the potential to yield even more advantages.

Soon Hoon et al. [14] established a PL-SLDV non-contact laser ultrasonic detection system to visualize cracks with a size of 10 mm × 10 μm. The state space attractor was reconstructed from the ultrasonic response, and the nonlinearity caused by damage was quantified using the nonlinear feature Bhattacharyya Distance. Later, using nonlinear Lamb wave mixing technology, a single 1.36 μm microcrack was detected by scanning the wave mixing area [29]. Shen et al. [68] analyzed the phenomenon of ultrasonic Lamb wave nonlinear scattering and mode conversion. The fatigue cracks around the rivet holes in an aluminum plate were detected by analyzing the energy of scattering higher-order harmonics. For anisotropic composite, Shen et al. [69] used the nonlinearity of the wavefield to detect delamination, and used the second-harmonic imaging algorithm to highlight the nonlinear interaction in the delamination region. The research indicates that nonlinear information in the wavefield is relatively insensitive to larger delamination.

In future research, further exploration of nonlinear information from laser ultrasonic Lamb waves could help detect early damage initiation more effectively.

### 4.5. Analysis of Comparison and Application

Table 1 lists the detection results from the literature related to LU-LDM, based on full wavefield data. This table focuses on the damage type, size, and assessment results. The specimens were mainly thin-walled aluminum alloy/CFRP structures. The thickness of the above structures ranged from 1 mm to 6 mm. The types of natural/artificial damage were mainly crack, groove and delamination with length and width distributed between 5–30 mm.

As shown in Table 1, NUT had the highest resolution for detecting damage at the micron level, but it was less sensitive to larger defects. LWE was considered the next best method and hac the ability to identify both the shape and depth of damage. The comparative analysis between several methods is shown in Table 2. AWPI and UFT methods enable real-time detection but require additional signal processing. The CCI method can automatically compensate for the Lamb wave dispersion effect, but its calculation is complicated, compared with other methods. Detection methods based on full wavefield data are not ideal for large-scale detection, but are well-suited for high-precision detection of small areas after the damage has been located.

## 5. Detection Method Based on the Small Amount of Wavefield Data

### 5.1. Sparse Distribution

#### 5.1.1. Geometric Positioning Method

By analyzing the group velocity and TOF of the scattered wave packet, it is possible to obtain the linear distance from the measurement point to the damage. Multiple distances obtained from multiple measurement points can be used to determine the damage’s location. The geometric positioning method (GPM) can roughly locate the damage by intersecting multiple geometric curves. GPM is classified into three main types, based on the type of geometric curve: triangular positioning method, elliptical positioning method, and hyperbolic positioning method. Researchers used the triangular positioning method for detection on a 10 kW composite wind turbine blade [49], drawing circles from two directions and using the intersection of multiple circles to successfully identify delaminations with ϕ10 mm.

**Figure 9:**
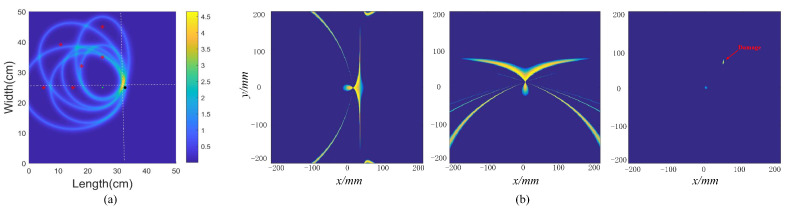
Geometric localization method based on laser ultrasonic Lamb wave. (**a**) Elliptical localization method [70]. (**b**) Hyperbolic localization images in both directions, and damage localization results after multiple image fusion [72].

In order to perform detection on an aluminum plate shell in the sandwich structure, Sikdar et al. [70] successfully detected hidden impact damage and holes using the elliptical positioning method, based on sparse wavefield data, as shown in Figure 9a. The results of the study showed that the distribution of measurement points has a significant impact on the accuracy of localization. The traditional multi-curve intersection approach needs to provide accurate damage locations. Therefore, an imaging algorithm, based on the GPM, was developed to assign magnitude values to each coordinate point, which can improve the recognition of damage. Yao Chen et al. [71] combined the ellipse location method and probability imaging algorithm to realize the location of the rectangular through a hole in the aluminum plate. Han et al. [72] proposed a hyperbolic positioning method, based on phase inversion theory, which does not need to consider dispersion effects and is not affected by material anisotropy. As shown in Figure 9b, this study achieved the localization of simulated damage in orthotropic composites.

GPM is a simple method belonging to the forward-solving algorithm, which does not require the complex inversion of the fluctuation equation. By sensing a small amount of wavefield data from a sparse/array distribution, the GPM can be used as a solution for rapid damage location. Despite its advantages, the GPM strongly depends on the accuracy of TOF, and dispersion effects can strongly influence the accuracy of this method.

#### 5.1.2. Damage Detection Based on Compression Sensing

The Compressed Sensing (CS) algorithm can under-sample wavefields at a rate lower than the spatial Nyquist sampling rate, while retaining the necessary information. It is characterized by the ability to reconstruct a high-dimensional signal from low-dimensional sampled data [73]. The spatial Nyquist sampling rate limits the scan interval of the array elements to less than half a wavelength of the highest frequency. By exploiting the sparsity of the wavefield under various bases, the CS can achieve dispersion curve reconstruction, de-dispersion, wavefield reconstruction, and damage localization [74].

**Figure 10:**
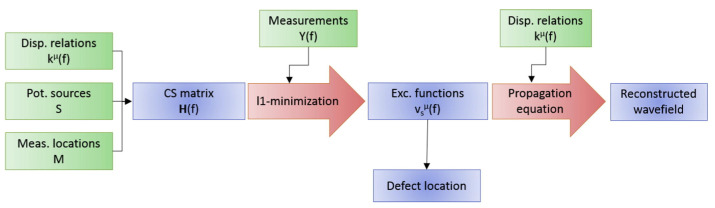
Sparse reconstruction process of wavefield based on CS [76].

If the damage location is taken as the detection target, the CS equation of the ultrasonic Lamb wave needs to be reconstructed, and the sparsity directly related to the damage exploited. The rapid development of LU-LDM, based on CS theory, comes after the relative maturity of laser ultrasonic technology. The laser can be subject to precise deflection, and facilitates the jitter acquisition of sparse wavefields. Esfandabadi et al. [75] sparsely represented the wavefield under different sparse representation bases (e.g., multidimensional Fourier transform, wavelet) and compared the variability of the reconstructed wavefield in different representation bases to localize multiple damage types in plates. In addition, the guided wavefield is represented as a linear superposition of a finite number of scattering source functions, according to the sparsity of the damage in the spatial location. Mesnil et al. [76] collected the wavefield data in the local region by laser ultrasonic jitter sampling. He used the *f*-domain wavefield expression to construct the higher-order CS equation. Finally, he achieved high-resolution imaging of the delamination in a GFRP plate with a sampling rate of 90%. The wavefield reconstruction process is shown in Figure 10.

Using the same principle, Li PF [77] analyzed guided wave wavenumber in anisotropic composite plates. The wavenumber dictionaries in different directions were constructed for wavefield reconstruction. Finally, the damage localization in CFRP was realized with a 86% sampling rate. In previous studies, only the phase information of the dispersion relationship was considered in the construction of the dictionary, and the amplitude modulation (e.g., source and sensor response, attenuation) was not considered. Moreover, amplitude modulation of broadband laser ultrasonic Lamb wave signal in low-frequency band increases the difficulty of signal interpretation. Lin et al. [78] considered both amplitude modulation and phase information to enhance the consistency of the atoms in the dictionary with the original signal. Therefore, the positioning accuracy of composite surface damage was enhanced.

The outstanding advantages of the LU-LDM, based on CS, are the significant reduction of sampling points, saving measurement time and storage space, and precision that relies on an exact dictionary library generated by a rational model. Its limitation is that the distribution of sparse measurement points significantly influences the results, and the repeatability of localization results needs further improvement. The computational efficiency of the reconstructed wavefield is low, and only preliminary damage localization can be achieved at this stage.

### 5.2. Dense Array Distribution

#### 5.2.1. Phased Array Imaging

The phased array imaging (PAI) algorithm requires the acquisition of dense arrays of wavefields with array element spacing typically less than half a wavelength [30]. With the development of machine vision technology, as well as upper computer software, the designability of measurement point distribution has improved, which facilitates the successful development of laser ultrasonic PAI technology. PAI technology is based on the beamforming theory of array signal processing [79]. The corresponding algorithm is the DAS imaging algorithm in the *t*-domain. However, its hot zone at the damage is far more extensive than the actual size, due to the influence of the dispersion effect on longitudinal resolution. The lateral effect is due to the poor performance of beam directivity, resulting in poor angular resolution [80]. Therefore, the wave packet extension, caused by dispersion, can alleviate the problem of low longitudinal resolution by implementing a DAS algorithm in the *f*-domain based on a known dispersion curve [81]. Further, Yu [82] used the adaptive weighting function of minimum variance distortionless response (MVDR) beamforming theory to improve the angular resolution by increasing the weight of the incident direction and suppressing other directions.

The energy leakage of the side lobe and grating lobe is the main reason for the low angular resolution, and, to a large extent, the array parameters affect the array beam directivity, where the topology is an important factor affecting the array performance [83]. With the high designability of the measuring points, the damage resolution can be improved by optimizing the array parameters. Ambrozinski et al. [84,85] compared the beam directivity of multiple 2D topological arrays, such as a cross, spiral, circular, and square ring. Among them, the spiral array has the best directivity.

Increasing the incidence direction can effectively improve imaging resolution [86]. To this end, Tian et al. [87] used PZT incidents in four directions around the damaged region and used a fence array to acquire the wavefield. The densely distributed clusters of subwavelength corrosion pits were imaged. Finally, they could identify most pits, except for the central pit, using data fusion techniques. The Total Focus Method (TFM) is the PAI algorithm of the multi-transmission and multi-receiving modes, and its imaging principle is Equation (4) [88].

(4) $$I\left(x,y\right)=\left|\sum_{i=1}^{N}\sum_{j=1}^{N}s_{i,j(i\neq j)}\left(t_{i,j}\left(x,y\right)\right)\right|$$

where, $S_{i,j}$(x,y) is the signal excited by the *i*-th sensing point and received by the *j*-th sensing point. $t_{i,j}$(x,y) is TOF, which is the time when the excitation signal from the *i*-th sensing point passes through the space point (x,y) and then scatters to the *j*-th sensing point. Liu et al. [88] built a fully non-contact laser ultrasonic detection system based on the TFM method to achieve damage localization. Each array element is used for excitation and reception, and two laser deflection systems are required. The symbolic coherence factor was also proposed to narrow the damage location hot zone, and the localization precision was much higher than that of PAI with single excitation and multiple receivers.

Signal acquisition for laser ultrasonic PAI takes only a little time. However, numerous virtual scattering points must be assigned, and much time is spent on post-processing. Therefore, PAI does not meet the need for rapid damage detection within a large area. Although many studies are approaching the actual damage size. Intrinsically, the beamforming theory, based on PAI, cannot break the half-wavelength diffraction limit and is not an effective method for obtaining high-precision imaging.

#### 5.2.2. Multiple Signal Classification

Multiple signal classification (MUSIC) separates the signal and noise subspaces by eigen decomposition of the received data covariance matrix. The spatial spectrum estimation of the signal source is achieved by scanning each point in the space and using the orthogonality between the space spanned by the steering vectors and the noise subspace [89]. Spatial spectrum estimation and damage location are similar, and their target is finding sound sources [90]. According to extensive research, it is known that the MUSIC algorithm has been applied to impact source localization and ultrasonic Lamb wave damage detection [91]. Most studies have used contact sensing, and only a few have used laser ultrasonic technology. The related research has focused on the following issues:

(1) Many topological arrays are designed, such as line [91], cross [92,93], petal [94], bilinear [95], biflabellate [96], and sparse [97]. The near-field 2D-MUSIC algorithm is used to locate the damage. The scattering waves are described as spherical waves when the damage is located in the near-field range. The 2D-MUSIC algorithm has a 2D-steering vector, and the near-field spatial spectrum is estimated as in Equation (5) [91]:
  (5) $$P_{\mathrm{MUSIC}}\left(r,\theta \right)=\frac{1}{A^{H}\left(r,\theta \right)U_{N}U_{N}{}^{H}A\left(r,\theta \right)}$$
  where A(r,θ) is a two-dimensional steering vector, *r* and θ are the scanning distance and angle, respectively. $U_{N}$ denotes the noise subspace tensed by the matrix of eigenvectors corresponding to small eigenvalues. By varying *r* and θ to scan each spatial point, the peak point of the spatial spectrum corresponds to the impact source/damage.
(2) MUSIC assumes that the incident wave is a single-frequency continuous wave, but laser ultrasonic Lamb waves have broadband characteristics. Shannon wavelets are good at extracting the narrowband component [98].
(3) It is not easy to artificially set the threshold to select the eigenvalues of the covariance matrix. For this reason, researchers proposed Gerschgorin’s disc theorem, based on unitary transform, which can judge the number of wave sources more directly [91].
(4) When there are multiple damages close to each other, or the damage is close to the boundary, the scattering wave interference causes the singularity of the covariance matrix. Researchers introduced a spatial smoothing method to average the covariance matrix of multiple subarrays to obtain a smoothed covariance matrix [91]. This method was only used for linear arrays.

The time reversal method allows adaptive focusing of the ultrasonic Lamb wave in the presence of unknown propagation media and array sensors. The array response matrix is obtained by a full matrix acquisition method. Eigenvalue decomposition of the time reversal matrix enables super-resolution imaging. Time reversal with the MUSIC (TR-MUSIC) and decomposition of the time reversal operator (DORT) are typical subspace class algorithms, both of which use noise subspace and signal subspace to construct the space spectrum, respectively. Yun et al. [99] built a detection system for PL-PZT and proposed the modified TR–MUSIC, which uses moving time windows to establish the local spatial spectrum at different times and distances, so as to improve the image quality of multiple damages. However, the distance resolution is very low. Yuan et al. [100] used an L-shaped array of PZT-SLDV detection system to acquire the array response matrix and used DORT–MUSIC to image multiple damage sites within an aluminum plate, showing excellent localization accuracy.

MUSIC has excellent advantages in damage detection efficiency and angular resolution, and it can break the Rayleigh diffraction limit to identify two damages with less than half-wavelength spacing. It does not need a complex model, nor does it need to extract TOF, and only uses simple mathematical analysis to describe the geometric relationship between the wave source and the array. However, MUSIC is not suitable for signals with low SNR, nor can it identify damage details. In the next step of research, the direct spatial spectrum estimation of broadband laser ultrasonic Lamb wave signals is a development direction that needs attention.

#### 5.2.3. Ultrasonic Lamb Wave Tomography

The ultrasonic Lamb wave tomography (LWT) arranges a sensing array around the detection area and designs a multi-transmission and multiple-receiving paths to cover the detection area [101]. High-accuracy damage reconstruction is achieved, based on the direct wave, scattering wave and transmitted wave. LWT can be performed in standard parallel projection [102], sector projection [103], or cross projection [104]. With the development of LU, the sensing method of tomography imaging has gradually evolved from traditional contact to non-contact.

Tomography is divided into two categories: the first category is transmission tomography, which is based on the ray theory and ignores scattering effects. A transmission tomograph typically uses TOF, amplitude, and frequency shift as characteristic parameters to detect and image damage [105]. The resolution is determined by the size of the first Fresnel zone and the wavelength [106]. Typical methods are the RAPDI [107], conversion [108] and iterative [109]. The reconstruction algorithm for probability detection of damage (RAPID) uses probabilistic statistical techniques to analyze the same parameters for different paths. Cho et al. [110] used signal difference coefficient (SDC) as the damage index. A ring-shaped array was designed to detect multiple defects in a large plate. The method does not require interpretation of the physical meaning, allowing flexible design of topological arrays, and enabling fast and effective wavefield reconstruction.

**Figure 11:**
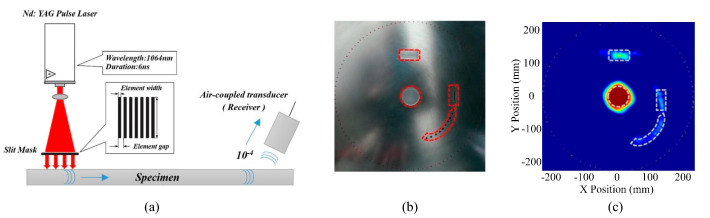
Robotic arm-assisted automated detection system and imaging results [111]. (**a**) PL-ACT tomography detection system. (**b**) Multi-damage sample. (**c**) Results of high-precision tomography imaging.

Cho et al. [111] proposed a Hilbert inverse projection algorithm, and constructed a robotic arm-assisted automatic detection system, as shown in Figure 11, which was successfully applied to detect different types of defects in aluminum plates with high accuracy. Hu et al. [112] used Lamb wave amplitude as the parameter, and used horizontal and vertical parallel projection to scan the detection area. A threshold was set to roughly evaluate the damage location. In subsequent research [109], the local area was scanned by cross-hole tomography, and the damages were reconstructed inversely, based on the least square method. Finally, high-precision reconstruction of notch and delamination in aluminum/composite hollow cylinders was achieved.

The second type is based on the scattering wavefield theory [113]. The fluctuation equations of the exact scattering wavefield are established, and the structure is reconstructed according to the measured external scattered waves [114,115]. The scattering waves carry comprehensive information and can truly reflect the distribution of the medium inside the structure. Huthwaite et al. [116] introduced the hybrid algorithm for robust breast ultrasonic tomography to the LWT. It provides accurate characterization of the remaining thickness of the plate. In addition, it can also improve the contrast of small-sized damage by iteration. Full waveform inversion (FWI) is a method, based on the iterative optimization calculation, which takes into account diffraction and high-order scattering effects and has the highest theoretical imaging resolution [117]. Various types of defects in complex structures can be detected, with defect detection resolution up to one-half wavelength. He et al. [118] combined the least squares reverse time migration and LWT. He built a circular array detection system, PZT-SLDV. Based on the born approximation, the scattering signals were used to reconstruct the damage shape, such as rectangles, and complex shapes, with multiple different sizes.

LWT has high hardware requirements, both exciting and sensing require devices to assist laser deflection. The acquisition efficiency is low, due to its having wraparound multi-transmission and multi-receiving. The imaging method of fluctuation equation inversion often has the advantage of high resolution, but the disadvantages of inefficient computing cannot be ignored.

### 5.3. Intelligent Detection Methods Based on Machine Learning

Machine learning (ML) is a data-driven decision-making method that automatically analyzes patterns from data and uses the patterns to make predictions about unknown data [119]. ML has a superior ability to identify and classify patterns in datasets and can be used as an extension of traditional damage detection techniques. Combining physical models with data models to establish a nonlinear mapping relationship between signal input and damage assessment can compensate for the shortcomings of traditional damage detection [120]. ML can be applied in several steps of ultrasonic Lamb wave damage detection, from the judgment of existence [121] to classification [122], localization [123], size assessment [124], depth reconstruction [125], and shape recognition [126]. The operational process can be summarized as obtaining detection information, extracting and selecting features, and classifying actual cases according to the categories that have been assigned labels. The signal is pre-processed by various algorithms and delivered to the training model as input. The following input forms are commonly used: original signal, parameters/images in the *t*-domain, *f*-domain or *t*-*f* domain, and low-resolution damage images.

Most machine learning methods, based on ultrasonic Lamb wave, use the convolutional neural network (CNN), and support vector machine (SVM), and a few use clustering in unsupervised learning [127,128]. Deep learning (DL) is a calculation method with a multi-layer neural network, which can solve the nonlinear mapping relationship between high-dimensional complex data, and it has been widely used in ultrasonic nondestructive testing in recent years [129]. The combination of DL and laser ultrasonic detection mainly focuses on the surface/subsurface damage of metal additive manufacturing structures, involving the damage classification and the assessment of depth and size [130,131,132]. However, research on combining DL with LU-LDM is still in a tentative stage, and only a few studies have had outstanding results in damage shape recognition. The DL is good at dealing with problems related to high-resolution imaging. H. Song et al. [133,134] constructed two multilayer convolutional neural networks with TFM/DAS imaging as the input and damage location and shape as the output. Their study successively achieved the localization and shape recognition of subwavelength damage. Moreover, the effectiveness of DL in high-resolution imaging of subwavelength damage was demonstrated. Chen et al. [80] applied clustering methods to damage localization and acquired wavefield arrays based on a fully non-contact laser ultrasonic detection method. The evolutionary strategy and clustering algorithm were combined to propose a search algorithm based on the symbolic coherence factor. The method improves the SNR, and the imaging time is less affected by the number of discrete grids.

ML is a mathematical tool connecting the input (measurement signals) and output (structural health states). The future trend is to exploit the correlation between ML and LU-LDM and to expand the database to enrich the training samples to achieve a leap forward in efficiency and accuracy.

### 5.4. Analysis of Comparison and Application

Table 3 lists the detection results from the literature related to LU-LDM based on a small amount of wavefield data. The studies were mainly on thin-walled aluminum alloy/CFRP structures and particularly honeycomb sandwich structures, whose thicknesses ranged from 1 mm to 10 mm. The types of damage were mainly holes, cracks, grooves, and delaminations, and partly by using magnets attached to the structure’s surface to simulate the damage. Most damage lengths and widths were distributed in the range of 5–30 mm, and there were also clusters of subwavelength damage and pitting damage of 2 mm.

The comparative analysis between several methods is shown in Table 4. GPA, CS, and MUSIC methods are suitable for rapid damage localization in a large area. The LWT can detect complex damage shapes with high resolution but requires multiple transmissions and receptions to construct a complete database. They are typically used after initial damage localization has been performed. A well-trained and sophisticated neural network allows for fast localization and quantification.

## 6. Combination of Multiple Methods to Balance Efficiency and Accuracy

Balancing detection efficiency and accuracy using a single detection method can be challenging. However, the fusion of multiple methods can help achieve a balance. Many researchers have developed a strategy of combining multiple methods, i.e., performing graded detection sequentially. This approach allows for rapid detection without sacrificing high-resolution results, making it a valuable tool in damage assessment. An overview of various combinations of LU-LDM are provided in Table 5.

(1) Combination of fast localization and high-precision imaging methods [135]. For damage detection over a large area, a small amount of wavefield data is first collected to roughly localize the damage. Then, a dense scan of the local wavefield is used to accurately quantify the damage. Combining multiple methods enables different methods to exert their advantages at different stages.
(2) The combination of CS and PAI imaging [136]. In a large area, sparse wavefield data are randomly collected, and the wavefield data of a dense array are reconstructed using the principle of CS. Based on this data, damage localization is achieved using PAI. This combination has a random wavefield acquisition, is not limited by the array, and saves many operations caused by reconstructing the full wavefield.
(3) Laser ultrasonic Lamb waves were combined with the laser ultrasonic body wave detection method [137]. The longitudinal waves penetrate the structure in a short time without dispersion [139,140,141]. The wavelength of longitudinal waves can reach the micron level and is more sensitive to damage in the thickness direction. A combination strategy involves using Lamb wave detection to obtain a rough location of the damage, followed by laser ultrasonic body wave imaging to achieve higher accuracy.
(4) Multiple CNN with different functions is constructed to achieve hierarchical detection [138]. In the outer loop, the damage detection model predicts the structure’s damage state (with or without). The inner loop predicts the damage’s location only when the outer loop’s detection result is present. This combination enables high-precision damage localization at the millisecond level.

## 7. Future Perspectives

The advantages of high designability of measurement points and high scanning precision have attracted more and more attention to LU-LDM. With the development of advanced laser and laser vibrometer technology, a framework of LU-LDM has gradually formed, and its effectiveness is reflected in the damage detection of thin-walled structures. Although some experimental achievements have been made, there is still much room for precision and efficiency. While an exhaustive list of future trends is not possible, an outlook on several potential future challenges and their corresponding developmental directions is provided.

(1) Promote the application of LU on actual project sites. Most detection objects in the existing studies are simplified to thin plates, but structures are complex, such as wing ribs, reinforcement bars, and screw holes. In the future, LU-LDM must solve real problems as the ultimate goal, build physical models of actual structures and study the corresponding detection methods.
(2) Research and development of a multi-channel laser ultrasonic testing system. The need for repetitive excitation and acquisition affects the improvement of detection efficiency. A laser vibrometer with multi-point synchronous acquisition can be developed to constitute a multi-channel laser ultrasonic detection system.
(3) Promote the application of laser ultrasonic modulation in time and space. Contact piezoelectric transducers can excite single-frequency continuous waves and narrowband signals. Compared with laser, its advantage lies in the strong selectivity of frequency and mode. The ultrasonic Lamb wave generated by a single laser spot is a broadband signal with no directivity. Optical devices, such as lenses and gratings, changing the shape of the laser spot, and, thereby, controlling the directivity and bandwidth of the ultrasonic wave.
(4) The existing research has focused on formed damage of centimeter size. However, the formation process is from initiation to evolution. Nonlinear ultrasound Lamb waves are susceptible to early microscopic damage of materials, such as fatigue cracks and creep holes. Therefore, using nonlinear LU-LDM to monitor material creep-damage and initiation-damage formation is a future development direction.
(5) Deep integration of laser ultrasonic detection methods with ML. At present, most methods are transplants of existing methods, which actually do not effectively utilize their specialties in solving nonlinear mapping relationships in damage detection. Future research must investigate the correlation between damage and signal features and build multi-level ML models between data and damage details.

## 8. Summary

This review systematically built a comprehensive framework for LU-LDM, which is expected to provide technical support to inspectors. Laser ultrasonic Lamb wave signals focus on the low-frequency A${}_{0}$ mode, which has the apparent disadvantage of a low SNR. Currently, most detectable damage sizes range from 5–30 mm, and include various types, such as corrosion, impact, delamination, and debonding. The summarization methods presented in this article can be applied to the online detection scenario, such as aircraft metal/composite skins, aircraft radar antenna radomes, high-speed train steel framework shells, and large ship metal decks.

According to different data characteristics and detection purposes, selecting the appropriate processing algorithms and detection methods can promote the development of LU-LDM from the laboratory to on-site applications. When high-precision imaging is necessary for local inspection, utilizing full-wavefield acquisition techniques, such as LWE, can be practical. It can achieve damage resolution at the micron level. If real-time imaging is necessary, AWPI and UFT can provide immediate graphical results, in tandem with detection. Alternatively, calculations with a small amount of wavefield data, such as MUSIC or trained neural network algorithms, can quickly infer the damage location. The former can quickly locate the damage location within a few seconds, but its distance accuracy may be limited. The latter option requires a significant amount of initial training. The combined use of multiple methods is the trend to improve overall detection efficiency and accuracy. As the cost of laser equipment continues to decrease, LU-LDM has the potential to become a detection technology that can replace contact sensing methods.

## Figures and Tables

**Table 1:** Laser ultrasonic detection results based on full wavefield data from the relevant literature.

Methods	Specimen	Damage Type and Size	Damage Information	Ref
WFA	Aluminum plate 1.27 mm	Narrow grooves 12.7 × 1.27 × 0.76 mm	Location	[52]
	Aluminum 1 mm	Triangular groove × 1/Letters bonded onto the surface × 3 10 mm/“USC” 18 × 20 mm	Location/Size/Shape	[53]
	Aluminum plate 6 mm	Corrosion × 4 30 × 30 mm	Location	[54]
LWE	CFRP 1 mm	Impact-induced delamination 32.3 × 22.6 mm	Location/Size/Shape 30 × 20 mm${}^{2}$	[57]
	Aluminum/CFRP plate 2 mm	Cut grooves 10 × 10 × 1/1.5 mm 20 × 20 × 1/1.5 mm	Location/Size/Shape	[58]
	Al/Al bonding plate 0.5/0.5 mm 0.5/1 mm	Delamination Φ30 mm	Location/Size/Shape	[59]
AWPI	CFRP composite wing 2.2 mm	Impact × 2 15.5 × 15.5 mm 26.6 × 22.9 mm	Location/Size 15.5 × 13.6 mm 21.7 × 19.8 mm	[1]
	Al-alloy tank liner 1.75–2.3 mm	Crack 11 mm	Location/Length 11 mm	[4]
CCI	Aluminum/Stainless- steel specimen 1 mm	Fatigue crack 5/10/30 mm	Location /Length 5.2/10.1/31 mm	[61]
	Stainless-steel plate 0.5 mm	Notches 40/60/90deg 10/3 × 2 × 0.25/0.1 mm	Orientation/Size	[44]
	CFRP plate 2.28 mm	Impact 17 × 22 mm	Location/Size	[62]
CSWE	Aluminum plate 4 mm	Notch 20 × 2 × 3 mm	Location/Length	[65]
	Aluminum plate 6 mm	Notch 20 × 2 × 3 mm	Location/Length 1.5 × 18 mm	[64]
UFT	CFRP radome 0.5 mm	Hidden circular impact Φ25 mm	Location/Size	[66]
	Aluminum plate 0.5 mm	Artificial/Closed crack 10 × 1 × 1 mm	Location/Length	[48]
NUT	Aluminum plate 0.5 mm	Crack 10 mm × 10 μm	Location/Size	[14]
	Aluminum plate 1 mm	Microcrack 1.36 μm	Location/Size	[29]
	two 2 mm thick quasi- isotropic CFRP plate bonded together	Delamination 20 mm	None	[69]

**Table 2:** Comparison of detection methods based on full wavefield data.

Methods	Detectable Information	Advantages	Disadvantages
LWE	Shape Depth	Enables high resolution imaging for damage detail	Sensitive to damage in the thickness direction only
AWPI	Size	Real-time detection with no conversion between spectral domains	Additional processing steps required in pre and post
CCI	Orientation Size	Automatic dispersion compensation	High calculation volume and low efficiency
CSWE	Size	Simple imaging principle	Highly influenced by standing waves, only centimetre-level resolution achieved
UFT	Size	Simple calculation, real-time detection, no conversion between spectral domains	Need to find the optimal damage correlation frequency
NUT	Size	Identification of pores or early sprouting microdamage	Special equipment is required to excite narrowband ultrasound

**Table 3:** Related literature and detection results of detection methods based on a small amount of wavefield data.

Methods	Specimen	Damage Type and Size	Damage Information	Ref
GPM	Wind turbine composite blade 3 mm	Delamination Φ10 mm	Location Error = 5.4 mm	[49]
	Aluminum Nomex sandwich core structure 1 mm	BVID(Impact damage)/hole Φ14/18/5 mm	Location	[70]
	Aluminum plate 1 mm	Rectangular through-hole damage 12 × 3 mm	Location	[71]
	Composite plate 1 mm	Through hole Φ10 mm	Location	[72]
CS	GFRP 1.6 mm	Delamination Φ25.4 mm between layer 2/3	Location/Size/Shape Compression ratios = 90%	[76]
	CFRP 1.6 mm	A circular magnet Φ10 mm	Location Compression ratios = 86% Error <2λ/3	[77]
	GFRP/CFRP plates 3.2/0.5 mm	Notch cut/Delamination 15 × 0.5 mm/Φ10 mm	Location/Size	[75]
	CFRP plate 2 mm	A square magnet 10 × 10 mm	Location	[78]
PAI	Aluminum plate 1 mm	Quartz rod × 4/Crack × 2 Φ4/6/8/10 mm 10 mm	Location/Orientation	[82]
	Aluminum plate 0.8 mm	Circular magnet/Through hole 25/20 mm 5 mm	Location/Size	[88]
	Aluminum plate 3.2 mm	Pitting corrosion cluster (3 × 3) Φ2 mm Interval:2 mm	Location/Size Except the center one	[87]
MUSIC	Aluminum plate 0.8mm	Through hole Φ10/20/30mm	Location	[93]
	Aluminum plate 2 mm	Two drilled holes Φ4 mm	Direction of Arrival	[99]
	T6061 aluminum plate 1.6 mm	Circular magnetic Φ6.35 mm	Location	[100]
LWT	Aluminum plate 3 mm	A round corrosion wastage Corrosion pits cluster 106 × 30 × 1 mm 32 × 100 × 1.5 mm	Location /Size	[110]
	Aluminum plate 1 mm	Rectangle/circle/microhole/strip 52 × 26/Φ52/Φ4/52 × 2 mm	Location/Size/Shape	[111]
	Aluminum plate 10 mm	Flat-bottom defects × 3 60 × 44 × 5 mm 42 × 30 × 4 mm 24 × 16 × 3 mm	Location/Size/Shape	[117]
	Aluminum alloy plate 2.29 mm	Rectangular/Complex shaped/ Multiple, varying-sized damage	Location/Size/Shape	[118]
ML	Aluminum alloy plate 1.6 mm	Crack-like defect 9.4 × 2 × 1.6 mm	Location /Size/Shape/ Orientation	[134]
	Aluminum alloy plate 1.6 mm	various subwavelength circular and notch-type defects 3/10/12/18 mm	Location /Size/Shape/ Orientation	[133]
	Aluminum plate 0.8 mm	Circular magnetic × 2 Φ25 mm	Location	[80]

**Table 4:** Comparison of detection methods based on a small amount of wavefield data.

Methods	Checkable Information	Advantages	Disadvantages	
GPM	Location	Simple principle, fast positioning	Accuracy affected by dispersion	
CS	Location	Breaks the Nyquist sampling limit	The construction of dictionary library has a great impact on the results	
PAI	Location	Simple principle with little data collection	Extremely inefficient point-by- point assignment calculations	
MUSIC	Location	Fast calculation and high resolution in orientation	Low SNR and dispersion result in low radial resolution	
LWT	Location Shape	High resolution	Multi-transmission and multi-receiving, low efficiency in acquisition	
ML	Location Shape	Fast classification/localisation, high accuracy quantification	Requires large amount of training data, poor physical interpretability	

**Table 5:** Multiple combinations of laser ultrasonic detection.

Step 1	Result from Step 1	Step 2	Result from Step 2	Ref.
PAI	Rough location	LWE	High-precision imaging	[135]
CS	Reconstruction of partial wave fields	PAI	Location	[136]
LWE	Damage imaging	Pulse echo method(body wave)	High-precision imaging	[137]
DL-CNN1	With or without damage	DL-CNN2	Location	[138]

## Data Availability

Not applicable.

## References

[1] Chia C.C., Lee J.R., Park C.Y., Jeong H.M. (2012). Laser ultrasonic anomalous wave propagation imaging method with adjacent wave subtraction: Application to actual damages in composite wing. Opt. Laser Technol..

[2] Papanaboina M.R., Jasiuniene E. The defect identification and localization using ultrasonic guided waves in aluminum alloy. Proceedings of the 2021 IEEE 8th International Workshop on Metrology for AeroSpace (MetroAeroSpace).

[3] Grammatikopoulos A. (2021). The effects of geometric detail on the vibratory responses of complex ship-like thin-walled structures. Mar. Struct..

[4] Choi Y., Lee J.R. (2017). Multi-directional adjacent wave subtraction and shifted time point mapping algorithms and their application to defect visualization in a space tank liner. Ndt E Int..

[5] Zhou B., Liu B., Zhang S. (2021). The advancement of 7xxx series aluminum alloys for aircraft structures: A review. Metals.

[6] Baker A.A. (2004). Composite Materials for Aircraft Structures.

[7] Jin H., Wang Y., Sun H., Li W., Qing X. (2022). Identification and imaging of multi-defects on a complicated composite structure by ultrasonic guided wave. Polym. Test..

[8] Gu G.H., Kim R.E., Kim E.S., Kim Y., Kwon H., Ahn S.Y., Seo M.H., Kim H.S. (2022). Multi-scale investigation on local strain and damage evolution of Al1050/steel/Al1050 clad sheet. J. Mater. Res. Technol..

[9] Khan A., Kim N., Shin J.K., Kim H.S., Youn B.D. (2019). Damage assessment of smart composite structures via machine learning: A review. JMST Adv..

[10] Dajani S.A.A.A., Dacus B.R., Dennett C.A., Burke M.G., Waldron L., Byun T.S., Wall J.J., Anglin K.B.-D., Dajani O.A.A.A., Krakowiak K.J. (2023). Detecting Thermally-Induced Spinodal Decomposition with Picosecond Ultrasonics in Cast Austenitic Stainless Steels. Acta Mater..

[11] Olisa S.C., Khan M.A., Starr A. (2021). Review of current guided wave ultrasonic testing (GWUT) limitations and future directions. Sensors.

[12] Mitra M., Gopalakrishnan S. (2016). Guided wave based structural health monitoring: A review. Smart Mater. Struct..

[13] Drain L. (2019). Laser Ultrasonics: Techniques and Applications.

[14] Liu P., Sohn H. (2016). Numerical simulation of damage detection using laser-generated ultrasound. Ultrasonics.

[15] Qiu J., Zhang C., Ji H., Tao C. (2020). Non-Destructive Testing for Aerospace Composite Structures Using Laser Ultrasonic Technique. Aeronaut. Manuf. Technol..

[16] Ma Y., Hu Z., Tang Y., Sheng Z., Ma S., Hu X., Luo W., Zeng Q., Guo L. (2020). Investigation of the mechanism and influence of laser wavelength and energy on laser opto-ultrasonic dual detection. Appl. Opt..

[17] Monchalin J. Non contact generation and detection of ultrasound with lasers. Proceedings of the 16th World Conference on Nondestructive Testing.

[18] Sun X., Tian Z., Lin B., Yu L. Guided wave damage detection with PZT-FBG sensing. Proceedings of the Nondestructive Characterization and Monitoring of Advanced Materials, Aerospace, and Civil Infrastructure 2016.

[19] Ahmed H., Mohsin A., Hong S.C., Lee J.R., Ihn J.B. (2021). Robotic laser sensing and laser mirror excitation for pulse-echo scanning inspection of fixed composite structures with non-planar geometries. Measurement.

[20] Monchalin J.P. (2004). Laser-Ultrasonics: From the Laboratory to Industry.

[21] James V., Carswell D., Riise J., Nicholson P.I., Graf N., Huber N., Gärtner M., Reitinger B., Scherleitner E., Burgholzer P. (2021). Robot Deployed Laser-Ultrasonic NDT System for Inspection of Large Aircraft Structures.

[22] Chen H., Liu Z. (2018). Application and Challenges of Signal Processing Techniques for Lamb Waves Structural Integrity Evaluation: Part B-Defects Imaging and Recognition Techniques. https://www.intechopen.com/chapters/62351.

[23] Rose J.L. Successes and Challenges in Ultrasonic Guided Waves for NDT and SHM. https://www.ndt.net/article/nde-india2009/pdf/1-PL-I.pdf.

[24] Lee J., Lee S.J. (2009). Application of laser-generated guided wave for evaluation of corrosion in carbon steel pipe. NDT E Int..

[25] Zhang C., Wang S., Guan X., Jin R. (2022). New Progress in Application of Laser Doppler Vibration Measurement Technology. Laser Optoelectron. Prog..

[26] Staszewski W.J., bin Jenal R., Klepka A., Szwedo M., Uhl T. (2012). A review of laser Doppler vibrometry for structural health monitoring applications. Key Eng. Mater..

[27] Mallet L., Lee B., Staszewski W., Scarpa F. (2004). Structural health monitoring using scanning laser vibrometry: II. Lamb waves for damage detection. Smart Mater. Struct..

[28] Ng K.M., Masurkar F., Peter W.T., Yelve N.P. (2019). Design of a new optical system to generate narrowband guided waves with an application for evaluating the health status of rail material. Opt. Lett..

[29] Sampath S., Sohn H. (2022). Non-contact microcrack detection via nonlinear Lamb wave mixing and laser line arrays. Int. J. Mech. Sci..

[30] Davis G., Rajagopal P., Balasubramaniam K., Palanisamy S., Nagarajah R. (2019). Laser Generation of Narrowband Lamb Waves for In-Situ Inspection of Additively Manufactured Metal Components.

[31] Davis G., Balasubramaniam K., Palanisamy S., Nagarajah R., Rajagopal P. (2020). Additively manufactured integrated slit mask for laser ultrasonic guided wave inspection. Int. J. Adv. Manuf. Technol..

[32] Ma Z., Yu L. (2019). Lamb wave defect detection and evaluation using a fully non-contact laser system. Proceedings of the Health Monitoring of Structural and Biological Systems XIII.

[33] Liu Z., Ma C., Chen H., Feng X., He C., Wu B. (2018). Comparison of Imaging Quality of Compact Rectangular Arrays Based on Laser and Piezoelectric Transducers. J. Beijing Univ. Technol..

[34] Gorgin R., Luo Y., Wu Z. (2020). Environmental and operational conditions effects on Lamb wave based structural health monitoring systems: A review. Ultrasonics.

[35] Ma Z., Yu L. Noncontact/remote material characterization using ultrasonic guided wave methods. Proceedings of the Smart Materials, Adaptive Structures and Intelligent Systems.

[36] Selim H., Moctezuma F.P., Prieto M.D., Trull J.F., Martínez L.R., Cojocaru C. (2019). Wavelet transform applied to internal defect detection by means of laser ultrasound. Wavelet Transform and Complexity.

[37] Lee J.R., Chia C.C., Shin H.J., Park C.Y., Yoon D.J. (2011). Laser ultrasonic propagation imaging method in the frequency domain based on wavelet transformation. Opt. Lasers Eng..

[38] Ding X., Li W., Xiong J., Shen Y., Huang W. (2020). A flexible laser ultrasound transducer for Lamb wave-based structural health monitoring. Smart Mater. Struct..

[39] Monchalin J.P. (1985). Optical detection of ultrasound at a distance using a confocal Fabry-Perot interferometer. Appl. Phys. Lett..

[40] Yi Z., Sanxing L. (2021). Study of Lamb Wave Dispersion Characterization Using Multiplexed Two-Wave Mixing Interferometer. Laser Optoelectron. Prog..

[41] Chia C.C., Jeong H.M., Lee J.R., Park G. (2012). Composite aircraft debonding visualization by laser ultrasonic scanning excitation and integrated piezoelectric sensing. Struct. Control. Health Monit..

[42] Chia C.C., Jang S.G., Yoon D.J. (2009). Structural damage identification based on laser ultrasonic propagation imaging technology. Proc. SPIE.

[43] Wang H., Zhang C., Ji H., Qiu J. (2022). Damage visualization using laser-generated residual guided waves with optimization of laser scanning path. Mech. Syst. Signal Process..

[44] Xiao W., Yu L. (2021). Thin-plate imaging inspection using scattered waves cross-correlation algorithm and non-contact air-coupled transducer. J. Nondestruct. Eval. Diagn. Progn. Eng. Syst..

[45] Dhital D., Lee J.R., Park C.Y., Flynn E. (2012). Laser excitation and fully non-contact sensing ultrasonic propagation imaging system for damage evaluation. Proceedings of the Industrial and Commercial Applications of Smart Structures Technologies 2012.

[46] Qing X., Li W., Wang Y., Sun H. (2019). Piezoelectric transducer-based structural health monitoring for aircraft applications. Sensors.

[47] Song P., Liu J., Liu L., Wang F., Sun X., Liu Z., Xu L. (2022). Contactless inspection of CFRP artificial disbonds using combined laser thermography and laser ultrasonics with optical microphone. Compos. Struct..

[48] Dhital D., Lee J.R. (2012). A fully non-contact ultrasonic propagation imaging system for closed surface crack evaluation. Exp. Mech..

[49] Park B., Sohn H., Malinowski P.H., Ostachowicz W. Damage detection in composites by noncontact laser ultrasonic. Proceedings of the EWSHM-7th European Workshop on Structural Health Monitoring.

[50] Ruzzene M. (2007). Frequency–wavenumber domain filtering for improved damage visualization. Smart Mater. Struct..

[51] Wang Z., Fei Y., Li B., Zhou A., Gorgin R. (2022). Research on the fk Domain Multimodal Damage Detection Imaging Fusion Method in Metal Plate. Trans. Indian Inst. Met..

[52] Michaels T.E., Ruzzene M., Michaels J.E. (2009). Incident wave removal through frequency-wavenumber filtering of full wavefield data. Aip Conf. Proc..

[53] Ma Z., Yu L. (2021). Lamb wave imaging with actuator network for damage quantification in aluminum plate structures. J. Intell. Mater. Syst. Struct..

[54] Jeon J., Jung H., Park G., Kang T., Han S. Laser-Scanning Based Damage Visualization Using Phase-Arrayed Local Wave Field Measurements. https://www.ndt.net/article/apwshm2018/papers/186.pdf.

[55] Flynn E.B., Chong S.Y., Jarmer G.J., Lee J.R. (2013). Structural imaging through local wavenumber estimation of guided waves. Ndt E Int..

[56] Truong T.C., Lee J.R. (2018). Thickness reconstruction of nuclear power plant pipes with flow-accelerated corrosion damage using laser ultrasonic wavenumber imaging. Struct. Health Monit..

[57] Tian Z., Yu L., Leckey C., Seebo J. (2015). Guided wave imaging for detection and evaluation of impact-induced delamination in composites. Smart Mater. Struct..

[58] Zhang H., Liang D., Rui X., Wang Z. (2021). Noncontact damage topography reconstruction by wavenumber domain analysis based on air-coupled ultrasound and full-field laser vibrometer. Sensors.

[59] Gao T., Liu X., Zhu J., Zhao B., Qing X. (2021). Multi-frequency localized wave energy for delamination identification using laser ultrasonic guided wave. Ultrasonics.

[60] Zhu R., Huang G., Yuan F. (2013). Fast damage imaging using the time-reversal technique in the frequency–wavenumber domain. Smart Mater. Struct..

[61] Xiao W., Yu L., Joseph R., Giurgiutiu V. (2020). Fatigue-crack detection and monitoring through the scattered-wave two-dimensional cross-correlation imaging method using piezoelectric transducers. Sensors.

[62] He J., Yuan F.G. (2016). A quantitative damage imaging technique based on enhanced CCRTM for composite plates using 2D scan. Smart Mater. Struct..

[63] He J., Yuan F.G. (2017). Lamb wave-based BVID imaging for a curved composite sandwich panel. Aip Conf. Proc..

[64] An Y.K., Park B., Sohn H. (2013). Complete noncontact laser ultrasonic imaging for automated crack visualization in a plate. Smart Mater. Struct..

[65] Zhang C., Ji H., Qiu J., Wu Y. (2014). Research on Interference Energy Calculation Method inLaser Ultrasonic Technique. Acta Opt. Sin..

[66] Chia C.C., Lee J.R., Park C.Y. (2012). Radome health management based on synthesized impact detection, laser ultrasonic spectral imaging, and wavelet-transformed ultrasonic propagation imaging methods. Compos. Part B Eng..

[67] Lissenden C.J. (2021). Nonlinear ultrasonic guided waves—Principles for nondestructive evaluation. J. Appl. Phys..

[68] Shen Y., Cen M., Xu W. (2019). Scanning laser vibrometry imaging of fatigue cracks via nonlinear ultrasonic guided wave scattering and mode conversion. Proceedings of the Health Monitoring of Structural and Biological Systems XIII.

[69] Shen Y., Cen M. Delamination Detection in Composite Plates Using Linear and Nonlinear Ultrasonic Guided Waves. Proceedings of the ASME International Mechanical Engineering Congress and Exposition.

[70] Balasubramaniam K., Sikdar S., Fiborek P., Malinowski P.H. (2021). Ultrasonic guided wave signal based nondestructive testing of a bonded composite structure using piezoelectric transducers. Signals.

[71] Yao C., Chen H., Li L. (2017). NDT Method for Thin Plate Damage Based on Laser Vibrometer. Nondestruct. Test..

[72] Han W., Feng K., Yang H. (2022). Phase Reversal Method for Damage Imaging in Composite Laminates Based on Data Fusion. Appl. Sci..

[73] Di Ianni T., De Marchi L., Perelli A., Marzani A. (2015). Compressive sensing of full wave field data for structural health monitoring applications. IEEE Trans. Ultrason. Ferroelectr. Freq. Control.

[74] Sabeti S., Harley J.B. (2020). Spatio-temporal undersampling: Recovering ultrasonic guided wavefields from incomplete data with compressive sensing. Mech. Syst. Signal Process..

[75] Esfandabadi Y.K., De Marchi L., Testoni N., Marzani A., Masetti G. (2017). Full wavefield analysis and damage imaging through compressive sensing in lamb wave inspections. IEEE Trans. Ultrason. Ferroelectr. Freq. Control..

[76] Mesnil O., Ruzzene M. (2016). Sparse wavefield reconstruction and source detection using compressed sensing. Ultrasonics.

[77] Li P., Lu Y., Xu C. (2021). Lamb wavefield reconstruction and damage imaging of composite plate based on compressed sensing. Acta Mater. Compos. Sin..

[78] Gao F., Hua J., Zeng L., Lin J. (2019). Amplitude modified sparse imaging for damage detection in quasi-isotropic composite laminates using non-contact laser induced Lamb waves. Ultrasonics.

[79] Ambroziński Ł. (2013). Beamforming of guided waves. Adv. Struct. Damage Detect. Theory Eng. Appl..

[80] Chen H., Xu K., Liu Z., Ta D. (2022). Sign coherence factor-based search algorithm for defect localization with laser generated Lamb waves. Mech. Syst. Signal Process..

[81] Li F., Luo Y. (2022). Damage Imaging of Lamb Wave in Isotropic Plate Using Phased Array Delay and Sum Based on Frequency-domain Inverse Scattering Model. Nondestruct. Test. Eval..

[82] Tian Z., Howden S., Ma Z., Xiao W., Yu L. (2019). Pulsed laser-scanning laser Doppler vibrometer (PL-SLDV) phased arrays for damage detection in aluminum plates. Mech. Syst. Signal Process..

[83] Yu L., Tian Z. (2016). Phased array techniques for damage detection in aerospace structures. Structural Health Monitoring (SHM) in Aerospace Structures.

[84] Ambroziński Ł., Stepinski T., Uhl T. (2015). Efficient tool for designing 2D phased arrays in lamb waves imaging of isotropic structures. J. Intell. Mater. Syst. Struct..

[85] Ambrozinski L., Packo P., Stepinski T., Uhl T. (2012). Experimental comparison of 2D arrays topologies for SHM of planar structures. Proceedings of the Nondestructive Characterization for Composite Materials, Aerospace Engineering, Civil Infrastructure, and Homeland Security 2012.

[86] Li F., Luo Y. (2021). Total Focusing Method Damage Imaging in Frequency Domain Using Laser-Ultrasonic Lamb Wave Based on Time-domain Filtering in Multi-band. Acta Mech. Solida Sin..

[87] Tian Z., Ma Z., Xiao W., Yu L. (2020). Noncontact laser vibrometry-based fence-like arrays with wavefield filtering-assisted adaptive imaging algorithms for detecting multiple pits in a compact cluster. Struct. Health Monit..

[88] Liu Z., Chen H., Sun K., He C., Wu B. (2018). Full non-contact laser-based Lamb waves phased array inspection of aluminum plate. J. Vis..

[89] Yang H., Lee Y.J., Lee S.K. (2013). Impact source localization in plate utilizing multiple signal classification. Proc. Inst. Mech. Eng Part C J. Mech. Eng. Sci..

[90] Yuan S., Zhong Y., Qiu L., Wang Z. (2015). Two-dimensional near-field multiple signal classification algorithm–based impact localization. J. Intell. Mater. Syst. Struct..

[91] Zhong Y., Yuan S., Qiu L. (2014). Multiple damage detection on aircraft composite structures using near-field MUSIC algorithm. Sensors Actuators A Phys..

[92] Han J.H., Kim Y.J. (2015). Time–frequency beamforming for nondestructive evaluations of plate using ultrasonic Lamb wave. Mech. Syst. Signal Process..

[93] Liu Z.H., Su R.X., Zhang T.T., Yu G., He C.F., Wu B. Full laser-based Lamb waves array imaging based on the two-dimensional multiple signal classification algorithm. Proceedings of the 2020 IEEE Far East NDT New Technology & Application Forum (FENDT).

[94] Zhong Y., Yuan S., Qiu L. (2014). Omni-directional impact localization method on composite structure using plum blossom array. Acta Mater. Compos. Sin..

[95] Bao Q., Yuan S., Guo F., Qiu L. (2019). Transmitter beamforming and weighted image fusion–based multiple signal classification algorithm for corrosion monitoring. Struct. Health Monit..

[96] Fu T., Wang Y., Qiu L., Tian X. (2020). Sector piezoelectric sensor array transmitter beamforming MUSIC algorithm based structure damage imaging method. Sensors.

[97] Yang X., Wang K., Zhou P., Xu L., Liu J., Sun P., Su Z. (2022). Ameliorated-multiple signal classification (Am-MUSIC) for damage imaging using a sparse sensor network. Mech. Syst. Signal Process..

[98] Yuan S., Bao Q., Qiu L., Zhong Y. (2015). A single frequency component-based re-estimated MUSIC algorithm for impact localization on complex composite structures. Smart Mater. Struct..

[99] Fan S., Zhang A., Sun H., Yun F. (2021). A local TR-MUSIC algorithm for damage imaging of aircraft structures. Sensors.

[100] He J., Yuan F.G. (2016). Lamb wave-based subwavelength damage imaging using the DORT-MUSIC technique in metallic plates. Struct. Health Monit..

[101] Zhao X., Royer R.L., Owens S.E., Rose J.L. (2011). Ultrasonic Lamb wave tomography in structural health monitoring. Smart Mater. Struct..

[102] Hua J., Cao X., Yi Y., Lin J. (2020). Time-frequency damage index of Broadband Lamb wave for corrosion inspection. J. Sound Vib..

[103] Hua J., Lin J., Zeng L. (2015). High-resolution damage detection based on local signal difference coefficient model. Struct. Health Monit..

[104] Zielińska M., Rucka M. (2021). Imaging of increasing damage in steel plates using Lamb waves and ultrasound computed tomography. Materials.

[105] Hutchins D., Jansen D., Edwards C. (1993). Lamb-wave tomography using non-contact transduction. Ultrasonics.

[106] Belanger P., Cawley P. (2009). Feasibility of low frequency straight-ray guided wave tomography. NDT E Int..

[107] Sheen B., Cho Y. (2012). A study on quantitative lamb wave tomogram via modified RAPID algorithm with shape factor optimization. Int. J. Precis. Eng. Manuf..

[108] Nagata Y., Huang J., Achenbach J., Krishnaswamy S. (1995). Lamb wave tomography using laser-based ultrasonics. Review of Progress in Quantitative Nondestructive Evaluation.

[109] Liu Y., Zhou S., Ning H., Yan C., Hu N. (2019). An inverse approach of damage identification using lamb wave tomography. Sensors.

[110] Albiruni F., Cho Y., Lee J.H., Ahn B.Y. (2012). Non-contact guided waves tomographic imaging of plate-like structures using a probabilistic algorithm. Mater. Trans..

[111] Park J., Lee J., Le Z., Cho Y. (2020). High-precision noncontact guided wave tomographic imaging of plate structures using a DHB algorithm. Appl. Sci..

[112] Hu B., Hu N., Li L., Li W., Tang S., Li Y., Peng X., Homma A., Liu Y., Wu L. (2014). Tomographic reconstruction of damage images in hollow cylinders using Lamb waves. Ultrasonics.

[113] Zhang H.Y., Ruan M., Zhu W.F., Chai X.D. (2016). Quantitative damage imaging using Lamb wave diffraction tomography. Chin. Phys. B.

[114] Chen X., Michaels J., Michaels T. (2015). A Methodology for Estimating Guided Wave Scattering Patterns From Sparse Transducer Array Measurements. IEEE Trans. Ultrason. Ferroelectr. Freq. Control..

[115] Zhang J., Drinkwater B., Wilcox P. (2008). Defect characterization using an ultrasonic array to measure the scattering coefficient matrix. IEEE Trans. Ultrason. Ferroelectr. Freq. Control..

[116] Huthwaite P., Simonetti F. (2013). High-resolution guided wave tomography. Wave Motion.

[117] Rao J., Ratassepp M., Fan Z. (2016). Guided wave tomography based on full waveform inversion. IEEE Trans. Ultrason. Ferroelectr. Freq. Control..

[118] He J., Rocha D.C., Sava P. (2020). Guided wave tomography based on least-squares reverse-time migration. Struct. Health Monit..

[119] Schnur C., Goodarzi P., Lugovtsova Y., Bulling J., Prager J., Tschöke K., Moll J., Schütze A., Schneider T. (2022). Towards Interpretable Machine Learning for Automated Damage Detection Based on Ultrasonic Guided Waves. Sensors.

[120] Harley J., Sparkman D. (2019). Machine Learning and NDE: Past, Present, and Future.

[121] Melville J., Supreet A.K., Deemer C., Harley J. (2017). Structural Damage Detection Using Deep Learning of Ultrasonic Guided Waves.

[122] Zhang Z., Pan H., Wang X., Lin Z. (2020). Machine Learning-Enriched Lamb Wave Approaches for Automated Damage Detection. Sensors.

[123] Zhang S., Li C.M., Ye W. (2021). Damage localization in plate-like structures using time-varying feature and one-dimensional convolutional neural network. Mech. Syst. Signal Process..

[124] Miorelli R., Fisher C., Kulakovskyi A., Chapuis B., Mesnil O., D’Almeida O. (2021). Defect sizing in guided wave imaging structural health monitoring using convolutional neural networks. NDT E Int..

[125] Liu B., Tang L., Wang J., Li A., Hao Y. (2014). 2-D defect profile reconstruction from ultrasonic guided wave signals based on QGA-kernelized ELM. Neurocomputing.

[126] Cui R., Azuara G., Scalea F., Barrera E. (2021). Damage imaging in skin-stringer composite aircraft panel by ultrasonic-guided waves using deep learning with convolutional neural network. Struct. Health Monit..

[127] Roy S.K., Chang F., Lee S.J., Pollock P., Janapati V. (2014). A novel machine-learning approach for structural state identification using ultrasonic guided waves. Safety, Reliability, Risk and Life-Cycle Performance of Structures and Infrastructures.

[128] Sattarifar A., Nestorović T. (2022). Emergence of Machine Learning Techniques in Ultrasonic Guided Wave-based Structural Health Monitoring: A Narrative Review. Int. J. Progn. Health Manag..

[129] Rautela M., Gopalakrishnan S. (2021). Ultrasonic guided wave based structural damage detection and localization using model assisted convolutional and recurrent neural networks. Expert Syst. Appl..

[130] Zhixiang X., Shouyan G., Fan Y., Lianfu L. (2021). Laser ultrasonic surface defects detection method based on 2D-CNN. J. Appl. Opt..

[131] Liu Z., Hu Z., Wang L., Zhou T., Chen J., Zhu Z., Sui H., Zhu H., Li G. (2021). Effective detection of metal surface defects based on double-line laser ultrasonic with convolutional neural networks. Mod. Phys. Lett. B.

[132] Guo S., Feng H., Feng W., Lv G., Chen D., Liu Y., Wu X. (2021). Automatic Quantification of Subsurface Defects by Analyzing Laser Ultrasonic Signals Using Convolutional Neural Networks and Wavelet Transform. IEEE Trans. Ultrason. Ferroelectr. Freq. Control..

[133] Song H., Yang Y. (2020). Super-resolution visualization of subwavelength defects via deep learning-enhanced ultrasonic beamforming: A proof-of- principle study. NDT E Int..

[134] Song H., Yang Y. (2020). Noncontact super-resolution guided wave array imaging of subwavelength defects using a multiscale deep learning approach. Struct. Health Monit..

[135] Tian Z., Yu L., Leckey C. (2016). Rapid guided wave delamination detection and quantification in composites using global-local sensing. Smart Mater. Struct..

[136] Song H., Yang Y. (2022). Accelerated noncontact guided wave array imaging via sparse array data reconstruction. Ultrasonics.

[137] Spytek J., Mrówka J., Pieczonka L., Ambroziński L. (2020). Multi-resolution non-contact damage detection in complex-shaped composite laminates using ultrasound. NDT E Int..

[138] Rautela M., Senthilnath J., Moll J., Gopalakrishnan S. (2021). Combined two-level damage identification strategy using ultrasonic guided waves and physical knowledge assisted machine learning. Ultrasonics.

[139] Lee Y.J., Hong S.C., Lee J.R., Hong S.J. (2021). Corner inspection method for L-shaped composite structures using laser ultrasonic rotational scanning technique. Adv. Compos. Mater..

[140] Lee Y.J., Lee J.R., Ihn J.B. (2018). Composite repair patch evaluation using pulse-echo laser ultrasonic correlation mapping method. Compos. Struct..

[141] Zhang K., Li S., Zhou Z. (2019). Detection of disbonds in multi-layer bonded structures using the laser ultrasonic pulse-echo mode. Ultrasonics.

